# Concordance of the cardiovascular patient education with the principles of Andragogy model

**DOI:** 10.1186/s13690-021-00763-5

**Published:** 2022-01-04

**Authors:** Negin Niksadat, Sakineh Rakhshanderou, Reza Negarandeh, Ali Ramezankhani, Ali Vasheghani Farahani, Mohtasham Ghaffari

**Affiliations:** 1 Department of Public Health, Faculty of health, Tehran Medical Sciences, Islamic Azad University, Tehran, Iran; 2 Department of Health Education and Promotion, School of Public Health & Safety, Shahid Beheshti University of Medical Sciences, Tehran, Iran; 3 Nursing and Midwifery Care Research Center, School of Nursing and Midwifery, Tehran University of Medical Sciences, Tehran, Iran; 4 Cardiac primary prevention research center, Tehran Heart Center, Tehran University of Medical Sciences, Tehran, Iran

**Keywords:** Patient education, (Cardiovascular disease) CVD, Andragogy

## Abstract

**Background:**

Patient education is a critical aspect of patient care and is considered a vital part of self-care (especially in patients with cardiovascular disease (CVD)) and health promotion. The literature supports incorporating the principles of the andragogy model (adult learning) into patient education. This study aimed to determine the concordance of the CVD patient education with the principles of the andragogy model.

**Methods:**

This cross-sectional survey was conducted on 384 adult CVD patients from 2 selected hospitals of Tehran. The sampling method was convenient, and the data collection tool was a researcher-made questionnaire based on the principles of the andragogy model. Data were analyzed using SPSS16 statistical software.

**Results:**

The mean age of the patients was 55.69 ± 13.01 years old. Frequency of distribution of the patients who, in total, selected the items of 4 or 5 for respecting the principles of andragogy model was as follows: 68.16% for the motivation, 66.29% for the need, 66.03% for the orientation, 54.16% for the experiences, 51.55% for the self-concept, and 44.65% for the readiness principle.

Also, three principles of motivation (77.37) need (74.97), and orientation (74.78) had the highest mean, respectively, in terms of adhering to this model. But the most common problems in patient education were related to the principles of readiness (64.35), self-concept (68.19), and experiences (77.71) with the lowest mean.

**Conclusions:**

The findings of this study provided valuable information on the flaws in patient education, including ignoring and disrespecting the principles of adult education. Correcting these detected defects and providing feedback to health professionals can improve the quality of patient education programs and patient satisfaction. Also, it empowers healthcare providers, patients, and families through effective education strategies.

## Background

Cardiovascular diseases (CVDs) are a group of disorders of the heart and blood vessels; therefore, preventing from them and also managing them are the priorities of national health [1]. World Health Organization (WHO) statistics in 2021 showed that cardiovascular diseases are the leading cause of death globally. 17.9 million people die each year from CVDs, an estimated 32% of all deaths worldwide. Over three quarters of CVD deaths occur in low-income and middle-income countries [1]. In Iran, the rate of deaths from cardiovascular disease has been reported to be 350 per 100 thousand people [2].

Education is an essential key for better coping with these diseases [3] Patient Education (PE) can be defined as “the process by which health professionals convey information to patients that can alter health behaviors or promote the medication adherence of the patients” [4]. PE is a critical aspect of patient’s care and it is considered one of the essential aspects of self-care and health promotion [5].

Concerning the outcomes of meta-analysis studies, among the CVD patients, PE can result in self -care behaviors development and also lead to the improvement of life quality associated with health, and along with that, also brings the potential decrease in healthcare costs and the acute events reappearance [6]. Moreover, PE is considered a high-priority component of the central and guideline standard care with CVD patients [7]. Although PE was recognized as a standard of care in the hospital to raise the understanding, and also to prepare the patient for discharge, it is a complicated issue because of the following matters: the teaching timing, staffing ratios, communication meanwhile education, and obstacles of information recalling [8].

With respect to several studies, the PE situation was not desirable in Iran. Consequently, PE program was applied imperfectly and unusually [9–11]. Those Barriers associated with the health care system, could contain the reduced length of hospital stay and also the reduced chances and time for instructing the patients [8]. Several factors can be considered as barriers to health care suppliers that, one by one could bring a negative effect on PE. Accordingly, applying paternalistic teaching style, lack of counseling skills, and ignoring the principles of adult learning and education are some of these barriers [8, 12–15]. Therefore, the most important barriers in education are recognized to be the deficiency in knowledge amongst health professionals (including nurses) in the field of educational requirements and PE methods [8, 11–14].

**Fig. 1:**
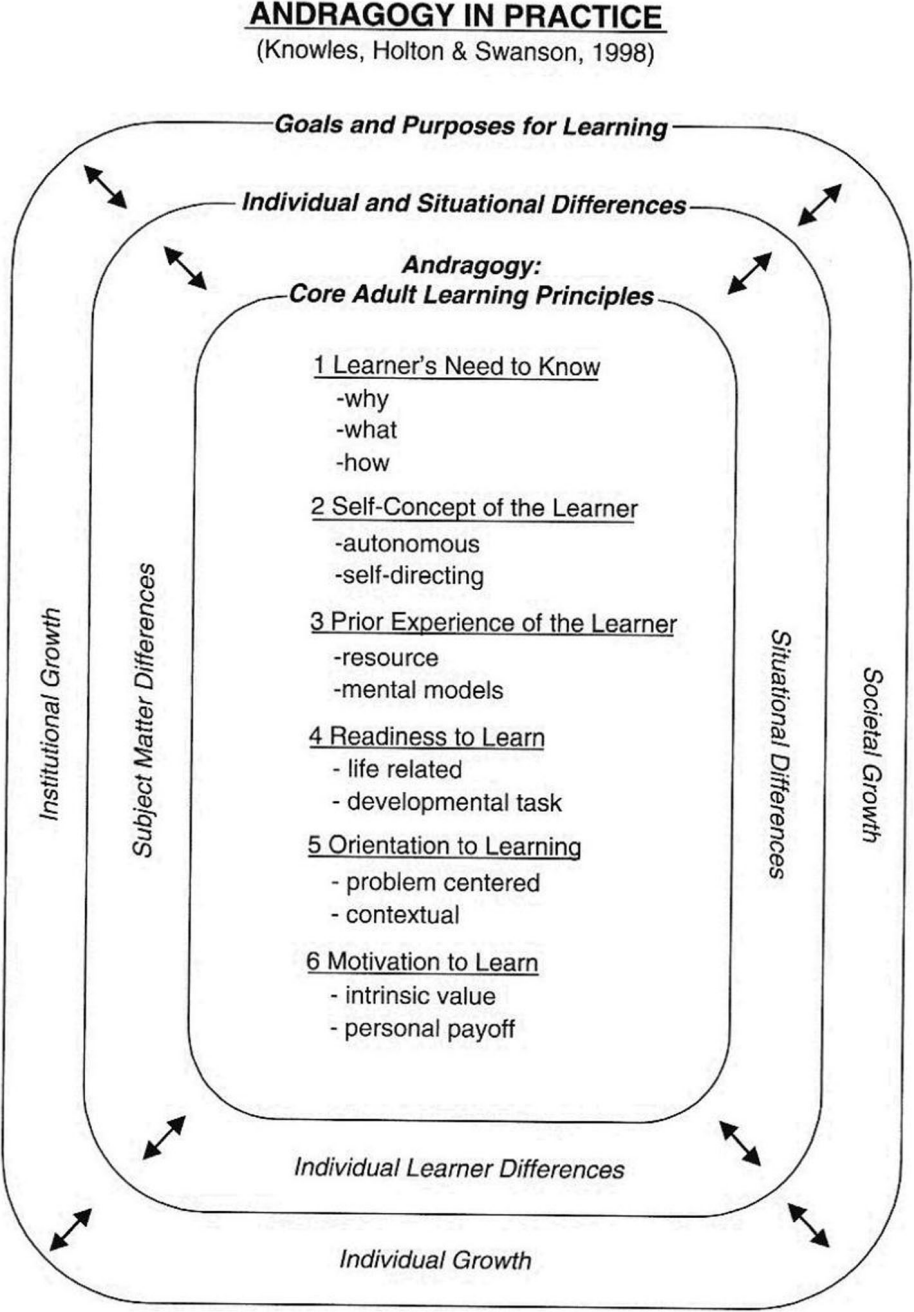
Andragogy in practice [5, 18]

There are different approaches to PE. Evidence shows that educational interventions which are planned based on the principles of adult learning and individualized patient-centered approaches are more effective [8, 16]. Andragogy has appeared to be one of the prevailing outlines and maybe the best-known adult learning theory over the past 40 years [17]. This model has been introduced as the science and art of helping adults learn. It holds that adults have different learning needs than children [5, 18]. Andragogy is a learning- centric conceptual framework that focuses on adults as self-directed learners and consists of six principles [5, 18]. These principles of Knowles’s model of andragogy contrast with pedagogy, a teacher-centered approach [19] and are as follows: adults need to understand why they need to know something (need to know), adults want autonomy over what to learn and move toward greater self-directedness in learning (self-concept) (self-concept), adults use prior their experience as a rich source of learning (prior experience), adults need to be ready for learning. It is closely linked to the need to know (readiness for learning), adults have problem-centered approach to learning that is based on immediate application of learning in real life (orientation to learning), Adults also are motivated to learn more by internal factors rather than external factors (motivation for learning) [5, 18] (Fig. 1).

In fact, studies have indicated that, the principles of andragogy can be applied by the health care providers such as nurses [20]. Considering these principles in the PE procedure provides health professionals with a greater knowledge on adult patients’ learning requirements, and helps them plan more effective PE interventions and improve learning outcomes [5, 16]. Also, it delivers a basis for enhancing the teaching instruments mainly designed for adult postpartum patients [5]. Starting education with holistic and patient-centered assessment of prior experiences and learning needs enable nurses to build on those previous experiences and support patients to merge new knowledge and skills. Active learning engages patients to be independent in solving their problems after discharge. Moreover, studies demonstrated that, regularly using these principles can have a positive effect on the patient’s agreement with the recommendations [21].

However, as most patients with CVD are adult, developing interventions based on the principles of andragogy can improve the quality of education and facilitate the attainment of health promotional and educational aims in this population. There is little evidence that the andragogy principles have been effectively applied to guide PE, especially among hospitalized patients with CVD [8].

Also, performing a preliminary study on the present situation is needed to identify problems in the PE process and develop appropriate future interventions. So, according to the limitations of the studies on applying the principles of andragogy for the PE among patients with CVD, this study was conducted to narrow this gap. The study aimed to determine the concordance of the CVD patient education with the principles of the andragogy model. The results of this research can be helpful to design and improve PE interventions in hospitals and health centers.

## Methods

The current study is a cross-sectional survey that aimed to determine the concordance of the CVD patient education with the principles of andragogy model**.** The sample of this research consists of 384 adult patients with CVD who were hospitalized or were referred to the 2 selected hospitals of Tehran (Modarres & Heart center hospital of Tehran. They were selected to the study if they were hospitalized or had the history of hospitalization, had the experience of receiving PE, and agreed to share their experiences of PE. Exclusion criteria included the patient’s unwillingness or inappropriate mood to complete the questionnaire, incomplete completion of the questionnaire or lack of sufficient experience of the PE process.

Since there is no information on this issue, in order to determine the sample size, the maximum value of 0.5 was considered for p and q. So, the sample size was calculated as 384 patients by considering α = 0.05, d = 0.05, and z = 1/96.

In this regard, a hospital was selected from each of the specialized heart hospitals of Tehran, Shahid Beheshti, and Iran University of Medical Sciences. Due to lack of cooperation, Heart Hospital of Iran University of Medical Sciences did not enter the study.

Then, the adult CVD patients in the selected hospitals were selected by the convenience sampling method to fill the sample size. It should be noted that, during the sampling process we attempted to consider the maximum variation by taking different background variables into account including participants’ age, gender, marital status, and educational level.

### Data collection

Data were collected over a period of about 8 months from September 2019 to April 2020. The data collection tool was a researcher-made questionnaire (10) based on the principles of the andragogy model in relation to evaluate PE among patient with CVD.

The development and psychometric process of the questionnaire was carried out in 2 steps. First, after the literature review on andragogy, adult learning theory and PE, an initial structured instrument was generated. The psychometric properties of the instrument were determined by evaluating the content validity ratio (CVR) and content validity index (CVI) by an expert panel review (*n* = 11). The Face validity was assessed through 10 patient interviews, the construct validity was tested through performing exploratory and confirmatory factor analysis (EFA) & (CFA), and the internal consistency of the questionnaire was checked by calculating Cranach’s alpha. (By a sample of 300 hospitalized adult CVD patients were selected through a convenient sampling method).

The EFA indicated the existence of 6 factors that accounted for 53.37% variance of the questionnaire. The principal component analysis using varimax rotation reduced the number of items from its original 59 to 40 items. The CFA showed a good fit of the data. Also, acceptable results for the internal consistency with Cronbach’s alpha value (0.72 to 0.93) was indicated. Finally, the questionnaire including six factors with 40 items was approved [10].

This Likert scale measurement instrument consisting of 8 items on demographic information and 40 items in terms of the six principles of andragogy model in PE was as follows: need to know (5 items) with scores of 5 to 25, prior experience of learners (9 items) with scores of 9 to 45, self-conception of the learners (9 items) with scores of 9 to 45, readiness to learn (5 items) with scores of 5 to 25, orientation to learning (5 items) with scores of 5 to 25, and motivation (7 items) with scores of 7 to 35. Each of the 40 items was scored on a 5 optional Likert-scale (1 = *strongly disagree;* 2 *= disagree;* 3 *= neutral;* 4 *= agree;* 5 = *strongly agree*). To avoid inducing the answers; some of the questions were reversely designed and were then scored.

During the data collection phase in this study, the questionnaire was completed by 384 patients who were enrolled as the real samples of the research. This process was held in patients’ hospitalization rooms or in a room in inpatient hospital clinics. The patients were asked whether the principles of andragogy were applied in their education.

### Data analysis

All data analyses were performed by SPSS 16 (Statistical Package for Social Sciences). Data were analyzed using descriptive statistics (frequency and mean and standard deviation) and analytical statistics tests (Spearman correlation coefficient). The significance level was set as *p* < 0.05.

#### Ethical considerations

The Ethical Review Committee of the Shahid Beheshti University of Medical Sciences (SBMU) approved the study (Code: IR.SBMU.PHNS.REC.1395.34). In addition to this, we obtained permission from authorities in the hospitals. Also, informed consent was obtained from each participant before the actual data collection. The consent was verbally obtained as we thought the patients might otherwise feel uncomfortable. To fulfill that, the researcher introduced herself and the study’s objectives. The participants were ensured the confidentiality of their names and other information. They were also informed of their liberty to leave the interview at any stage. This was considered to be an acceptable consent procedure by the ethics committees both at institutional (Shahid Beheshti University of Medical Sciences) and national level (Ministry of Health) for exceptional situations, and was particularly confirmed for this study.

## Results

A total of 384 CVD patients; 58.9% of whom were males and 41.1% were females, participated in this study. The mean age of the participants was 55.69 ± 13.01 years old. Descriptive statistics for the participants of the study are shown in Table 1.

**Table 1:** Demographic features of the respondents to the questionnaire

	Frequency	Percent
**Gender**		
**male**	**226**	58.9
**female**	**158**	41.1
**Marital Status**		
**married**	**341**	88.8
**Single**	**38**	9.9
**others**	**5**	1.3
**Education**		
**junior school**	**149**	38.8
**High school**	**144**	37.5
**Academic**	**91**	23.6
**Job**		
**unemployed**	**35**	9.1
**worker**	**25**	6.5
**Employee**	**31**	8.1
**Self-employment**	**83**	21.6
**retired**	**124**	32.3
**Housekeeper**	**85**	22.1
**Residence**		
**Tehran province**	**222**	57.8
**Other Province**	**161**	41.9
**CVD disease**		
**Coronary artery**	**208**	54.2
**congenital**	**30**	7.8
**HF**	**28**	7.3
**other**	**118**	30.7

### The principle of the need to know

According to this principle of the andragogy model, adult patients need to know why they need to know something, before they learn and do it. They want to know how the experience of learning will be helpful for them [18].

This principle from the andragogy model was measured using the Likert scale in the questionnaire containing five questions. Most of the patients (more than half) had a positive point of view on the observance and attendance to this principle in educations. Accordingly, in this case, 66.29% of them chose items 4 and 5 for the questions of the need (Table 2).

**Table 2:** Frequency distribution of participants related to the observance of the six principles of the andragogy model

Principle	Score of 5		Score of 4		observance of principle	
	Frequency	Percent	Frequency	Percent	Frequency	Percent
Need to know	991	32.25	1046	34.04	2037	66.29
Prior experience	1127	22.57	1577	31.59	2704	54.16
self-concept	1134	19.68	1836	31.87	2970	51.55
orientation to learning	502	26.14	766	39.89	1268	66.03
readiness for learning	520	13.54	1195	31.11	1715	44.65
motivation	961	31.28	1133	36.88	2094	68.16

Moreover, this principle was one of the three principles whose means were more than other principles (74.97 out of 100) (Table 3).

**Table 3:** Distribution of participants regarding to the observance of the principles of andragogy model based on the mean ± SD

Principle	Mean	SD	Score range of each principle	Mean score of 100
Need to know	29.98	4.36	8–40	74.97
Prior experience	46.65	8.27	13–65	71.77
self-concept	51.14	9.16	15–75	68.19
orientation to learning	18.69	4.15	5–25	74.78
readiness for learning	32.17	6.7	10–50	64.35
motivation for learning	30.95	6.58	8–40	77.37

Standard deviation (SD)

Questions 2 and 4 had the maximum percentage of agreement with the observance of need to know principle in education, and 84.9% of the patients stated that, the educator explained them before the education that they can take care of themselves based on these educations, and 84.7% of the patients stated that, these educations were useful to solve the problems related to their diseases.

### The principle of prior experiences of the learner

One of the important principles in adult education is to attend to the learner’s prior experience [18]. This principle from the andragogy model was measured using the Likert scale questionnaire containing nine questions. Approximately half of the patients had a positive point of view on the observance and attendance to this principle in educations, and 54.16% of the patients chose the items of 4 and 5 for the questions related to this principle (Table 2). However, in general, this principle was one of the three principles with less mean than other principles of the model (71.77 out of 100) (Table 3).

The maximum percentage of agreement with the observance of the experience principle was related to the following options: “ I felt that, educator can understand my concerns and problems related to my diseases” (65.9%), “my previous experiences on my disease helped me to better learn the educations” (63.5%), and “educator spent enough time to educate me” (63.3%). These options obtained the maximum percentages of agreement and complete agreement with the observance of (respect to) this principle. In addition, in response to the question: “educator used some sentences and words, which were not understandable”, 65.1% of the patients believed that this principle was observed in the educations by choosing the option of disagreement.

### The principle of self-concept of the learner

According to this principle of the andragogy model, adult patients imagine that they are responsible for their decisions, so this is an important issue for them to have the right to choose in education and learning [5].

This principle was measured using the Likert scale questionnaire containing nine questions. Approximately half of the patients had a positive point of view on the observance and attendance to this principle in educations, and 51.55% of the patients chose the items of 4 and 5 for the questions related to the self-concept (Table 2). However, in general, this principle was one of the three principles with less mean compared to the other principles of the model (68.19 out of 100) (Table 3).

The maximum percentage of agreement with the observance of (respect to) this principle (scores of 4 and 5) was related to the following options: “during the educations, it was determined that what responsibilities I have, regarding my disease”, “educator gave me an opportunity to ask my question”, and “ I have better understood the educations due to participating in the education process”. Based on these options, 72.6, 67, and 60.4% of the patients believed this principle was observed in education, respectively. Moreover, regarding the option of “the behavior of the educator caused me to have much more anxiety”, 63.1% of the patient believed that, this principle was observed during the education by choosing the option of disagreement.

The maximum percentage of disagreement with the principle was related to the following option: “they asked my opinion about what method should be used or how the method should be implemented (for instance, they speak with me or give me educational CD) (43.3%), which obtained the maximum percentages of disagreement and complete disagreement. In addition, in response to the option of “if educational CD and movie were used to give me the education, I could learn more better”, the total number of the patients who believed that the self-concept principle was not considered and observed was more (52.1%).

### The principle of the orientation to learning

One of the principles in adult education is the orientation and tendency of the adults to learn, which is based on the fast and immediate application and emphasizes the current and real condition of life [18]. This principle from the andragogy model was measured using the Likert scale questionnaire containing five questions.

Most of the patients had a positive point of view on the observance and attendance to the principle of the orientation to learn in the educations, and believed that the educations were applicable. In this case, 66.03% of the patients chose agreement and complete agreement options (scores 4 and 5) for the questions related to the orientation to learning (Table 2). In general, this principle was one of the three principles with a higher mean compared to the other principles of the model (74.78 out of 100) (Table 3).

Two questions of “ The education prepared me to perform the duties and responsibilities I had with my self-care (75.5%)” and “information obtained in this education is in the form, which can be immediately used in my life and in handling affairs related to my disease” (73.1%), obtained the maximum percentages of agreement and complete agreement related to the observance of this principle (score 4 and 5).

The most percentage of disagreement with the observance of this principle was related to the following question of “educator asked me to speak about the problems related to the care of myself or think that I am exposed to them”.

### The principle of readiness

One of the important principles in educating adult patients is attention to readiness and their learning preferences [18]. This principle from the andragogy model was measured using the Likert scale questionnaire containing five questions.

Approximately less than half of the patients had a positive point of view on the observance and attendance to the principle of the readiness and its observance in the educations. In this case, 44.65% of the patients chose the items of (scores of) 4 and 5 for the questions related to readiness (Table 2). In general, this principle was one of the three principles with a lower mean compared to the other principles of the model (64.35 out of 100) (Table 3).

In total, the maximum percentage of positive responses to the observance and attendance to this principle (scores 4 and 5) was related to the options of “ during the education, the room was so crowded and noisy that I could not understand or focus on the education” and “I did not understand the education because most cases were educated during the delivery”. In this case, 51.8 and 51% of the patients believed that their readiness was considered for the education by choosing the disagreement option.

The maximum percentage of the responses believed to no attention to this principle was related to the options of “I was not ready to understand the issues that were presented in the education” and “The environment in this education was not suitable for learning”.

### The principle of motivation

According to this principle of the andragogy model, adult patients are more motivated by internal causes and motivators compared to external ones [5]. This principle of the andragogy model was measured using the Likert scale questionnaire containing seven questions.

Most of the patients had a positive point of view on the observance and attendance to the principle of motivation and its observance in educations. They were encouraged to learn through internal incentives. In this case, 68.16% of the patients chose items 4 and 5 for the questions related to motivation (Table 2). In general, this principle had the highest mean compared to the other principles of the model (77.37 out of 100) (Table 3).

Relationships of education with the patient’s problems (75.3%) and a good sense of education (76.3%) were the most frequent motivations for learning. Most cases most cases of disagreement with the principle of motivation were motivation to learn because of the patient opinion in teaching and admiration for learning from the educator.

## Summary

In this study, patients’ experiences were assessed in terms of whether the PE was in line with the principles of the andragogy model.

Findings revealed that the principles of adult education are not sufficiently respected in education for adult patient with cardiovascular diseases. Observing the frequency distribution of patients’ responses in relation to the considering the principles of this model in education, generally indicated that most common problems in PE were related to the principles of readiness (44.65%), self-concept (51.55%), and experiences (54.55%), respectively. Also, these three principles, in the same way, had the lowest mean score in terms of considering the principles of this model (64.36, 68.19 & 77.71). On the other hand, the percentage of the patients who chose the items (scores) of 5 and 4 for the principles of the motivation (68.16%), need to know (66.29%), and the orientation (66.03%) was higher than the other three principles from this model. In addition, these tree principles had the highest means respectively (77.37, 74.97, and 74.78).

## Discussion

This study was the first attempt to determine the compliance of the CVD patient education with the principles of andragogy with using a self-made questionnaire based on principles of this model. According to the results of the present study, the principles of adult education are not sufficiently considered in PE for adult Patients with CVD. The most common problems were related to three principles of readiness, self-concept, and the experience with the least average and frequency in terms of attention in educations.

### The principle of the need to know

In the present study, the majority of the patients acknowledged that that this principle was considered in educations. They understood the need to know educational content and found it useful. So that, the mean score related to the observance of this principle was higher than the other principles from the model. In line with these findings, in Zolezzi et al.’s study to develop and evaluate an educational program on CVD risk assessment based on the adult learning model, the learning needs of individuals in the educational process were considered and emphasized [22].

In the present study, more than half of the patients agreed that the educator explained the benefit of learning the subject before starting the education. A similar finding was found in a study by Day et al. on understanding the factors affecting the successful education of the postpartum discharged patients based on the andragogy model. More than half of the patients stated that the nurses explained learning educational materials and their benefits [5].

In general, according to Knowles’ model, starting a course without knowing what, why, and how the learning experience will be useful, can reduce the motivation and interest of adult learners [5, 18]. However, some teachers stated that, they did not feel the need to provide these explanations, because they assumed that learners entered the classroom with this information [23].

### The principle of the learner’s self-concept

In our study, self-concept was one of the three principles that were less observed in PE. For example, regarding the role of the patient in the choice of method, materials, and timing of the education, a low percentage of the patients stated that, the educators attended to their points of views. Compared with other studies, a study based on adult learning principles, encouraged self-directed learning and created an atmosphere of trust and respect for patients by allowing them to set the time and place of PE [24]. Also, Hart et al. provided the possibility for the patients with breast cancer to choose their preferred method of obtaining information based on their preferences [25].

On the other hand, most of the patients in our study stated that, the educator would allow them to ask their questions. However, former research found the accessibility of nurses for addressing patients’ questions and concerns as one of the essential aspects of PE [26].

Another major problem mentioned by about half of the patients was not receiving educational videos or CDs and additional resources to learn independently by themselves. Kim et al. also emphasized the importance of introducing educational tools and resources to hospitalized patients, which can be used after returning home [21].

In accordance with this andragogy principle, using active learning and involving adult patients in their health care and education, also making them feel responsible for their learning, has been emphasized in many studies to improve the outcomes of education and treatment [3, 16, 18, 22].

In general, the patients’ failure to participate in education and lack of respect for the principle of self-concept can have many causes. For example, doctors and nurses may think that the patients have no adequate competence to decide or participate in education; they have a sense of superiority to the patients; due to a large number of the patients, the answers to their questions are limited. As in Roberts’ study, educators were skeptical of the learners’ ability in self-direct learning [23].

### The principle of readiness to learn

In this study, attention to patients’ readiness to learn received the least attention compared to other principles of andragogy.

There is no specific schedule for PE, and it is implemented at the time and conditions desired by the physicians or nurses. In most cases, patients’ readiness and their differences in physical, mental, and individual characteristics are not considered. For example, some patients may have pain during the education [5, 8]. This highlights the importance of personalizing the process of PE and avoiding providing patients with a lot of information when they are unable to understand [3]. Karen et al. required nurses to be aware of the patient learning barriers such as pain and employ strategies to manage their physical and mental problems before education. They emphasized that neglecting these barriers and patients’ readiness can negatively affect their learning [27].

On the other hand, Anxiety and stress from inappropriate environments impede learning and retaining information. Sandra et al. have considered maintenance of a proper learning environment necessary to promote learning [28].

### The principle of the orientation to learning

In our study, this principle was one of the three principles that were more observed in PE. The majority of the patients stated that the education prepared them for the tasks and responsibilities to take care of their illness. The information gained in this education was available in a way that could be immediately applied to life and being used in cases related to their illness. In consistent with our study, Day et al. also found that most nurses considered this principle of andragogy and provided practical education [5]. In another study, many patients with CVD believed that their education would be applicable to make a complete recovery.

According to the andragogy, adult patients’ orientation and tendency to learn is problem-based, based on the rapid and immediate application, and emphasizes the current and real-life situations [18]. Therefore, health care providers should train the patients in a practical and usable way in the patient’s life. Patients need more information at the time of discharge, especially on the medication use and the problems they may encounter after returning to home [28, 29].

### The principle of motivation

According to the results of this study, almost all patients had the necessary motivation to follow the education and were encouraged to learn through the internal incentives. Moreover, the score of this principle had the highest mean compared to other principles from the model. For example, the majority of the patients stated that they were encouraged to learn by understanding the role of education in helping them take care of themselves and not depending on others. They also cited the relevance of education to their problems and illness and the role of education in assisting them to make decisions on their problems as the factors to encourage learning. So, the relevance of the education to the patient’s problems was one of the two cases that had the highest frequency of motivation for learning. In consistent with our study, Day et al. also found that childcare and self-care were the most important factors in motivating learning [5].

The importance of paying attention to the principle of motivation in providing education and encouraging learning as the responsibility of the educator has been reported in numerous studies [28, 30, 31]. Adult patients need attention to be encouraged to learn [32]. Therefore, it is necessary and is recommended that, health care providers including physicians and nurses pay attention to motivate the patients, especially internal motivations, in providing educational programs.

### The principle of prior experiences of the learner

According to the andragogy model, having more and different quality experiences in adult patients cause a wider range of individual differences which should be considered in PE [16, 18].

In this study, the patients also had different perspectives regarding their conditions. However, this principle was one of the three principles that were not considered sufficiently in educations. For example, only about half of the patients stated that, they were asked about their knowledge and information on their illness as well as their level of education at the beginning of the education. Similarly, Hart et al. did not carry out an official evaluation on the level of education and health literacy of patients with breast cancer prior to education. So, patients are faced with a large amount of inadequate information [25].

According to the results of many studies, assessing learner knowledge and paying attention to the patients’ and their families’ literacy level is an essential factor in providing understandable discharge time education [19, 24, 33]. Also, Nurses should consider patients’ preferred learning styles and apply a combination of styles such as visual aids and spoken and written instruction to make patients more receptive [16, 24]. Mentrup et al., found that most of the verbal information provided for patients with CVD was vague and nonspecific [3].

In the present study, many patients stated that the educator could understand their concerns and problems of their illness. Also, more than half of the patients noted that the educator spent enough time teaching them. In line with these findings, Day et al. reported that most nurses understood the patients’ different concerns [5]. In another study, Bree et al. showed that, most physiotherapists were able to spend enough time for teaching each patient [32].

In general, an effective PE should start with holistic and patient-centered evaluation of prior experiences [16, 32]. A former study emphasized that trying to understand patients with CVD before education and providing individualized information will increase their satisfaction and prepare them for self-care after discharge [3].

### Strengths and limitations

The strength of this study is its novelty. It is the first study to determine the concordance of CVD patient education with the principles of the andragogy model. Also, it was the first attempt to use a self-made questionnaire based on principles of andragogy in this regard. Among the limitations of this study, the need to spend enough time by patients and complete the questionnaire carefully and find patients who have a good experience of received PE was more important.

## Conclusion

The findings of this study indicate that the principles of adult education are not sufficiently observed and respected in adult PE programs. The important reason for this problem may be the lack of knowledge of health care providers about the principles of the adult education model and its importance in PE.

Given that most cardiovascular patients are adults, it is essential to educate them on the principles of the andragogy model. Adherence to the principles of adult education in the planning of these educations can improve the quality of interventions and allow achieving the ultimate goals of PE.

So, evaluating the education provided by health education professionals, giving them feedback on existing deficiencies, and educating them on the principles of adult education and its importance, will enhance their performance in the PE process. Developing a consistent and specific model to design and deliver education to the patients can improve care quality and patient outcomes. Therefore, healthcare providers will be empowered; patient satisfaction will be improved, and, most importantly, effective education strategies will empower patients and families. It should be noted that the findings of this study can be applied to the educational process of other adult patients.

## Data Availability

The data used to support the findings of this study are available from the corresponding author upon request.

## Abbreviations

CVD: Cardiovascular disease
CVDs: Cardiovascular diseases
PE: Patient education
WHO: World Health Organization
SBMU: Shahid Beheshti University of Medical Sciences
SPSS: Statistical package for social sciences
CVR: Content validity ratio
CVI: Content validity index
EFA: Exploratory factor analysis
CFA: Confirmatory factor analysis
